# Aquatic environmental DNA detects seasonal fish abundance and habitat preference in an urban estuary

**DOI:** 10.1371/journal.pone.0175186

**Published:** 2017-04-12

**Authors:** Mark Y. Stoeckle, Lyubov Soboleva, Zachary Charlop-Powers

**Affiliations:** 1 Program for the Human Environment, The Rockefeller University, New York, New York, United States of America; 2 Laboratory of Genetically Encoded Small Molecules, The Rockefeller University, New York, New York, United States of America; University of Hyogo, JAPAN

## Abstract

The difficulty of censusing marine animal populations hampers effective ocean management. Analyzing water for DNA traces shed by organisms may aid assessment. Here we tested aquatic environmental DNA (eDNA) as an indicator of fish presence in the lower Hudson River estuary. A checklist of local marine fish and their relative abundance was prepared by compiling 12 traditional surveys conducted between 1988–2015. To improve eDNA identification success, 31 specimens representing 18 marine fish species were sequenced for two mitochondrial gene regions, boosting coverage of the 12S eDNA target sequence to 80% of local taxa. We collected 76 one-liter shoreline surface water samples at two contrasting estuary locations over six months beginning in January 2016. eDNA was amplified with vertebrate-specific 12S primers. Bioinformatic analysis of amplified DNA, using a reference library of GenBank and our newly generated 12S sequences, detected most (81%) locally abundant or common species and relatively few (23%) uncommon taxa, and corresponded to seasonal presence and habitat preference as determined by traditional surveys. Approximately 2% of fish reads were commonly consumed species that are rare or absent in local waters, consistent with wastewater input. Freshwater species were rarely detected despite Hudson River inflow. These results support further exploration and suggest eDNA will facilitate fine-scale geographic and temporal mapping of marine fish populations at relatively low cost.

## Introduction

Effective ocean management depends on knowledge of the diversity, distribution, and abundance of marine life. Because censusing marine life requires costly specialized equipment, skilled personnel, and time, sampling is rarely dense or frequent. As compared to surveying sessile species such as shellfish, monitoring fish and other nekton is particularly challenging because they move—in response to daylight, temperature, and season; to evade capture or predation; and in relation to other short and long-term factors.

Analyzing water for DNA traces shed by organisms—environmental DNA (eDNA)—may help people to learn affordably, quickly, and frequently about the presence and abundance of known forms of marine life, especially fish [1–3]. eDNA sensitivity, accuracy, distribution, and duration have been carefully studied in a number of controlled and natural freshwater settings [4–11]. The utility of eDNA in marine environments, vastly larger and more complex, is just beginning to be explored [12–16].

**Fig 1:**
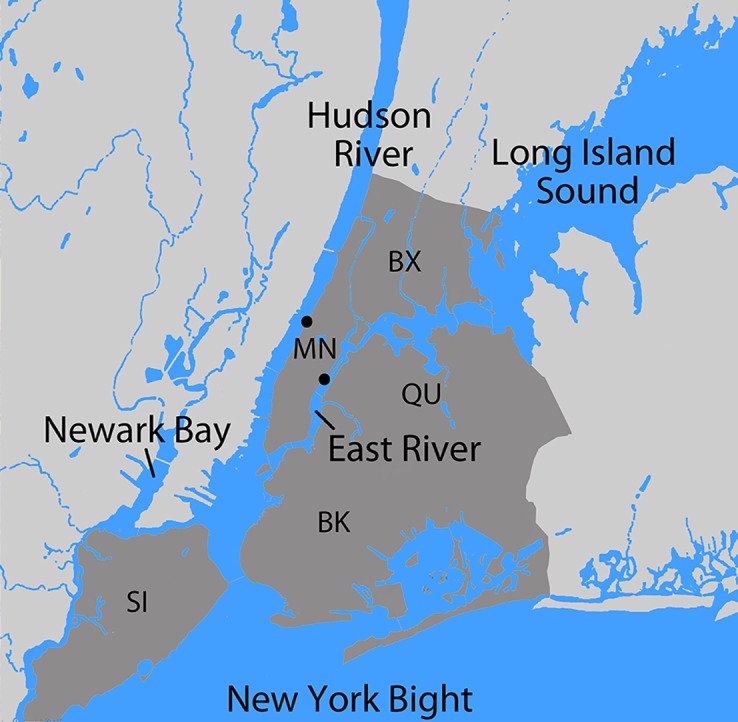
Lower Hudson River estuary. Major waterways are labeled, and New York City limits highlighted in dark gray with boroughs indicated [Bronx, (BX), Manhattan (MN), Queens (QU), Brooklyn (BK), Staten Island (SI)]. Sampling sites are marked by dots. Figure prepared using U.S.G.S. topographic maps as templates.

Here we tested the sensitivity and specificity of aquatic eDNA for fish detection in the lower Hudson River estuary surrounding New York City, the most heavily urbanized estuary in North America (Fig 1) [17]. This complex ecosystem receives daily freshwater inflow from the Hudson River and ocean tidal inflows from Long Island Sound and New York Bight. Although water quality has improved dramatically over the past few decades, fecal contamination from wastewater remains ubiquitous [18,19]. The estuary is essential habitat for anadromous fish that arrive from the ocean in the spring to breed, including American shad, blueback herring, striped bass, and the threatened Atlantic sturgeon, and the catadromous American eel, that returns to the Hudson after breeding in the mid-Atlantic [20].

The lower Hudson River estuary offers eDNA assessment advantages. First, multiple seining surveys document local fish populations. Second, large seasonal changes in fish abundance test eDNA temporal specificity. Third, daily freshwater and saltwater inflows, which might carry eDNA from non-resident species, challenge geographic localization of eDNA. Contrasting environments within the estuary query eDNA distribution at a finer scale. Finally, most resident fish have mitochondrial sequences in GenBank, enabling identification of amplified DNA sequences.

Aquatic eDNA is often applied to detect rare or well-hidden taxa not easily found by traditional methods, using species-specific primers or exhaustive amplification [21–25]. Here we aimed to assess all resident fish species—or at least all of the more common ones—utilizing a metabarcoding protocol. We analyzed samples collected at two contrasting estuary locations over six months. The overall hypothesis was that eDNA is an indicator of fish presence and abundance. More specifically, we hypothesized that local marine fish would be detected and freshwater and open ocean species would not; that detection would reflect local abundance as determined by a compiled checklist; that eDNA detections would differ by season consistent with the springtime movement of fish populations into regional inshore waters and estuaries; and that there would be differences among the sites related to what is known about fish habitat preferences. Our findings are largely consistent with these hypotheses. eDNA detected abundant and common estuary species more often than uncommon ones, rarely found freshwater species despite Hudson River inflow, differed by season consistent with the springtime movement of fish populations into regional inshore waters and estuaries, and differed by site consistent with fish habitat preferences. In addition, we found eDNAs of commonly consumed fish, which may reflect wastewater contamination in the estuary. We discuss limitations and aspects needing further study, and potential of this technology for marine fish assessment.

## Results

**Fig 2:**
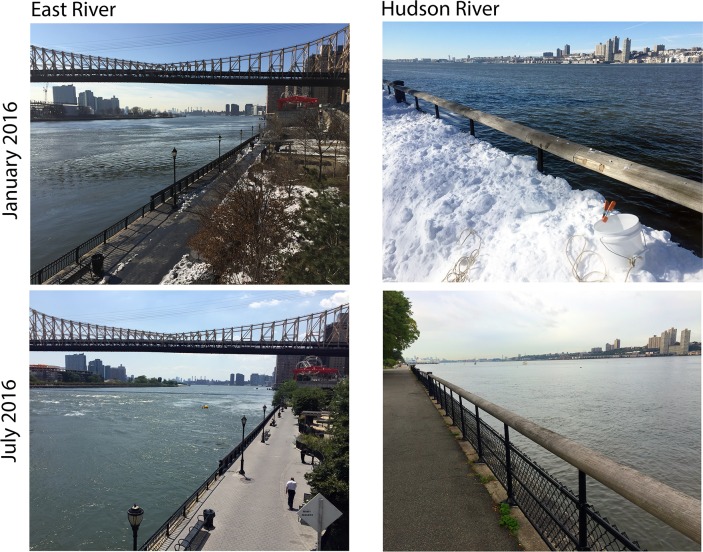
Collection sites, views south towards New York harbor. Collection equipment (bucket, rope) visible in upper right panel.

**Fig 3:**
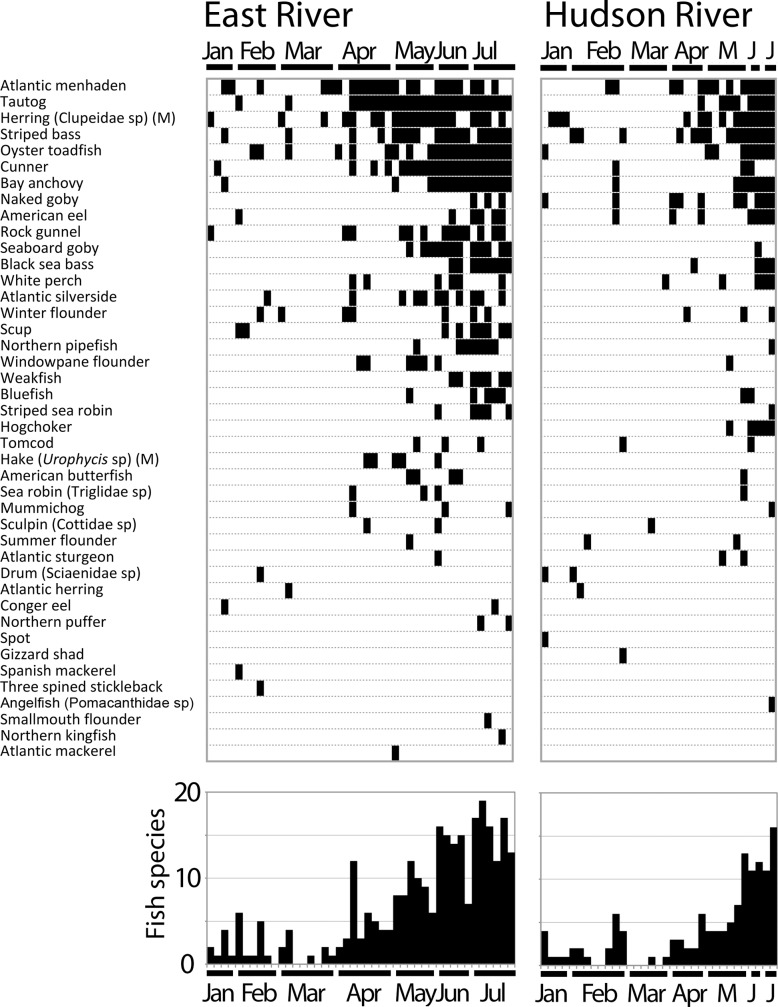
Local marine fish eDNA presence and absence by date and site. Top: black indicates eDNA presence; each column represents an individual sample, arranged by collection date, with collection site and month shown; each row represents a unique amplified fish sequence with identification at left, arranged by decreasing frequency of detection and number of reads. (M) indicates matching multiple local species; incomplete identifications are shown with genus or family names. Collection dates, scientific names, and identification details are in S3 and S4 Tables. Bottom: number of fish species detected per sample.

A checklist of 85 fish species was compiled from 12 local surveys [26–28], with species categorized according to the number of surveys in which they were present: abundant (9–12 surveys; 14 species), common (5–8 surveys; 28 species), or uncommon (1–4 surveys; 43 species) (S1 Table). We collected 76 one-liter shoreline surface water samples over six months beginning in January 2016. The two collection locations were a marine (high flow, near-ocean salinity, low turbidity) site on the East River and a more typical estuarine (low flow, brackish, high turbidity) site on the Hudson River (Figs 1 and 2). eDNA from water samples was amplified with vertebrate-specific 12S mtDNA primers [29] and sequenced on an Illumina MiSeq. Bioinformatic analysis of MiSeq fastq files detected eDNA from 51 fish species, most of which (82%) matched local marine taxa (S2 and S3 Tables). Consistent with eDNA detectability as reflecting local abundance, the assay identified most of the abundant or common checklist species and relatively few of the uncommon ones (81% vs. 23%, p = 2 x 10^−6^, Fisher’s exact test) (S2 Table). In addition, abundant or common species were found in more samples than were uncommon fish (average detections per species, 10 vs. 1, p<0.01, Mann-Whitney U test). Overall, nearly all reads (93%) matched locally abundant or common fish species. At both sites there was a strong seasonal increase in fish eDNA detection, consistent with the taxonomically widespread movement of fish populations into regional inshore waters and estuaries in the spring (Fig 3) [30–32]. Species seasonally detected by eDNA known to exhibit regional springtime population increases include Atlantic menhaden (*Brevoortia tyrannus*), Atlantic silverside (*Menidia menidia*), river herrings [alewife (*Alosa pseudoharengus*), American shad (*A*. *sapidissima*), blueback herring (*A*. *aestivalis*)], bay anchovy (*Anchoa mitchilli*), black sea bass (*Centropristis striata*), bluefish (*Pomatomus saltatrix*), cunner (*Tautogolabrus adspersus*), striped bass (*Morone saxatilis*), scup (*Stenotomus chrysops)*, tautog (*Tautoga onitis*), and weakfish (*Cynoscion regalis*).

**Fig 4:**
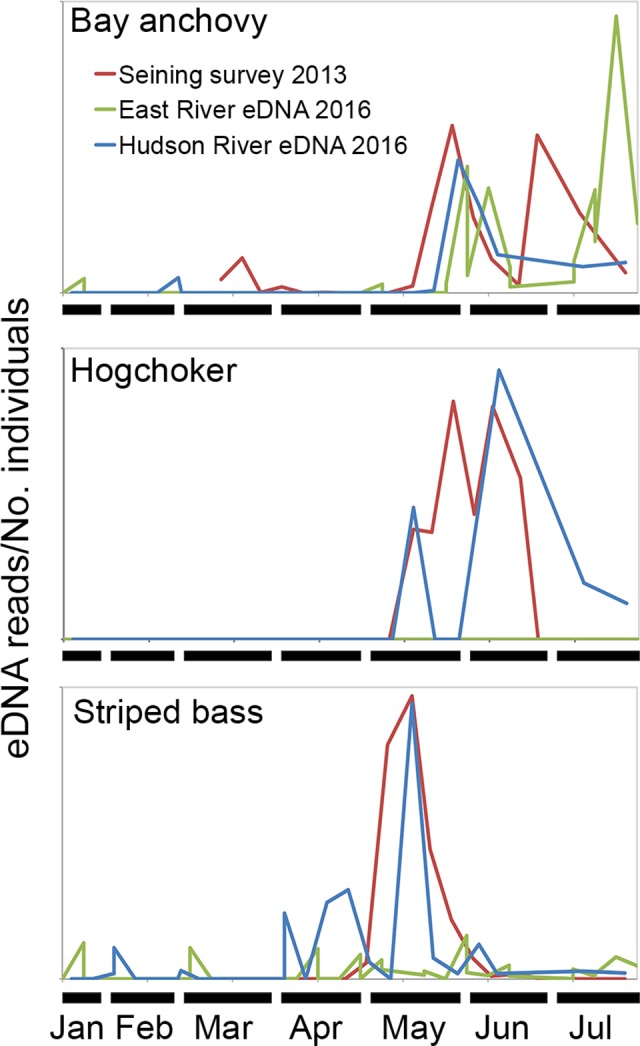
eDNA reads and seining survey abundance by date. Scales of eDNA reads and no. individuals differ between species. Number of individuals from 2013 Long River Survey trawls conducted between March 13 and July 27 at the southern tip of Manhattan, approximately 10 km by water from both study sites [28]. For bay anchovy and hogchoker, graph represents number of yearling and older individuals; for striped bass, graph represents number of eggs collected in the entire estuary as a proxy for migratory adult individuals, as the survey seining equipment does not capture mature striped bass.

This study employed a single amplification protocol. In pilot experiments, we found reproducibility was correlated with number of reads—more reads, more likely to be detected on repeat amplification (overall reproducibility 64%; for reads <1000, 1000 to 10,000, and >10,000, 41%, 63%, and 87%, respectively, all comparisons, p<0.001, Fisher’s exact test) (S5 Table). 12s eDNAs matching species with declining or threatened populations were observed, including American eel (*Anguilla rostrata*), Atlantic sturgeon (*Acipenser oxyrhinchus*), and winter flounder (*Pseudopleuronectes americanus*) (Fig 3). Local time series seining data was available for a few species; in these cases the timing of eDNA seasonal increases (by presence/absence and read number) paralleled increasing fish numbers in the estuary (Fig 4). Most species were more common at East River site or were similarly distributed; the two species more often detected at Hudson River site [naked goby (*Gobiosoma bosc*), hogchoker (*Trinectes maculatus*)] are estuary specialists (Fig 3) [30,33]. An interesting comparison is naked goby, largely detected in Hudson, and its congener seaboard goby (*Gobiosoma ginsburgi*), which was in East River samples only.

One unexpected result was amplification of eDNA matching locally rare or absent fish species, comprising nine (18%) of the 51 unique fish sequences (S2 Table). One or more exotics were detected in 7% of samples; in total they comprised 2% of fish reads. About half were menu or aquarium species including Atlantic salmon (*Salmo salar*), European sea bass or “branzino” (*Dicentrarchus labrax*), Pacific red snapper (*Lutjanus peru*), and common guppy (*Poecilia reticulata*). One sample amplified a sequence matching (100% identity) Pacific sand lance (*Ammodytes hexapterus*), and differing (98% identity) from the resident congener Atlantic sand lance (*A*. *americanus*). The remaining exotics were freshwater species resident in the Hudson River watershed—common carp (*Cyprinus carpio*), darter (*Ethiostoma* sp), channel catfish (*Ictalurus punctatus*), and white sucker (*Catostomus commersonii*).

## Discussion

Here we show that the detection of fish eDNA correlates with fish abundance as determined by a compiled checklist and differs by season consistent with the widespread movement of fish populations into regional inshore waters and estuaries in the spring. To our knowledge, this is the most extensive time series analysis of marine fish eDNA to date. Together with other studies, these results support eDNA for marine fish assessment and highlight limitations and aspects needing further study.

This study focused primarily on detection, i.e., presence or absence of eDNA. There was not sufficient data on either side of the potential correlation to assess eDNA reads as a general measure of abundance. First, published seining studies to date have not analyzed seasonal fish populations at the temporal and geographic scale of this manuscript. Most are not time series; most do not report number of individuals collected; none have been conducted in the East River. The one time series that reports on abundance of multiple species, the Long River Survey, uses equipment designed to capture ichthyoplankton—eggs and juvenile forms—and not adult fish. We were able to use this data for comparison to eDNA reads in a few species, either because adults are small and so are captured in survey nets (bay anchovy, hogchoker), or because the species’ natural history is well enough known to use eggs as a proxy for migratory adult individuals (striped bass). Second, the single amplification protocol employed in this study is insufficient to quantify differences between species.

Our single amplification protocol reproducibility was dependent on apparent eDNA abundance, as reported in other metabarcoding studies [34]. Overall reproducibility was relatively low, 64%, suggesting that single amplification was likely to miss about one-third of the species in a given sample. However, the composite sensitivity summing all samples should be high. This suggests additional samples or amplifications are unlikely to yield additional taxa at these particular estuary sites, unless there are late seasonal migrations not covered by this time series. Failure to detect was not due to our OTU screening procedure, as no fish species were entirely eliminated from a given MiSeq run by the thresholds applied. Nonetheless, we detected few of the uncommon estuary fishes. This may reflect the highly skewed distribution of fish abundances in the estuary. For example, in the 2013 Long River Survey, 10 of 61 estuary species accounted for 99% of individuals [28]. For scarce taxa, species-specific primers can be more sensitive than metabarcoding [35]. Besides rarity or absence of fish, failure to detect may be due to localization of eDNA within the estuary, primer mismatch, reduced shedding or increased turnover of eDNA, or other unknown factors [36,37].

In about 10% of samples we found DNAs matching fish that are commonly consumed but are locally rare or absent—Atlantic salmon (*Salmo salar*), Pacific red snapper (*Lutjanus peru*), and European sea bass (*Dicentrarchus labrax*). Commonly consumed Salmonidae are reported as an apparent contaminant in some marine eDNA studies [14,16]. We hypothesize that the menu species DNAs in this study originate from processed or raw sewage in local waters. Directly analyzing these sources will help evaluate this conjecture. If confirmed, this could limit study of commonly consumed species in environments with wastewater contamination. Other potential sources of apparently exogenous DNAs include laboratory procedures, commercial reagents, sequencing error, and undocumented haplotypes of local species [38,39]. In addition to fish eDNAs, human, domestic animal, and terrestrial wildlife eDNAs were commonly obtained (S6 Table), as routinely observed in aquatic eDNA studies.

*in silico* analysis confirmed highly conserved primer binding sites among local vertebrates for the 12S eDNA target, and new sequences reported in this study boosted target coverage to 80% of local fish. As expected, the short amplicon sequence matched multiple local species in some cases, limiting species resolution. Alternate targets may enable discrimination [39].

Contamination did not appear to be an issue (see METHODS for details), suggesting fish eDNA work can be safely performed in a benchtop setting with standard molecular biology methods, at least when assaying relatively common species. More advanced precautions, such as those used in ancient DNA investigations, may be needed in some circumstances, particularly if the aim is to detect very rare DNAs [16,34,40]. The workflow was relatively slow, on average about three months between collecting samples and obtaining sequence results, primarily because the fixed cost of a MiSeq run mandated accumulating multiple samples before submission. Sample collection aside, the entire process could be accomplished without stress with present technology in one week, and in about 24 hours if urgency demanded. Total direct costs excluding salaries of personnel were low, about $10,000.

This study demonstrates amplified aquatic eDNA correlates with fish abundance, seasonal movements, and habitat preference in an urban estuary with large fresh and saltwater inflows. Taking into account inherent limitations and multiple aspects needing further study, we and others anticipate that the relatively low cost of sampling, which can be performed by diverse persons with modest equipment, will facilitate surveys at much finer temporal and geographic scales than possible with traditional techniques [2,41–44]. Early applications in regional waters could include assessments of aquaculture on proximate marine ecosystems and when closures of dredging or fishing are needed to protect threatened migratory species. In environments lacking a comprehensive reference sequence library, e.g., in the deep sea and coral reefs, eDNA could at least offer a list of “dark taxa” [45] that inspires further exploration and efforts to capture specimens. Given the simplicity of collecting water samples, a wide variety of interested persons could participate in surveys [46]. Looking ahead, how best to assemble and display aquatic eDNA analyses generated by different researchers deserves attention. Portals that compile wildlife observations (e.g., FISHBase, MARCO, eBIRD) benefit both the public and science.

## Materials and methods

### Hudson River estuary checklist

A compiled occurrence list of 85 fish species was constructed from 12 local surveys conducted between 1988–2015 [26–28] (S1 Table). Chondrichthyans were excluded due to primer mismatch as described below. Species were classified according to number of surveys in which they were detected: abundant (9–12), common (5–8), or uncommon (1–4). Most (80%) of the 85 species have 12S target region sequences in GenBank or generated in this study (S1 and S4 Tables).

### Primer evaluation

We first tested whether a broad-range vertebrate 12S-V5 region primer pair [29] would amplify local freshwater and marine vertebrate species. *in silico* evaluation demonstrated highly conserved primer binding sites (S7 Table). Sharks and rays were an exception; most local species have a mismatch at the 5’ primer terminal nucleotide, likely to inhibit amplification. The amplified segment differs among most local species, allowing species-level identification. A pilot study (23 samples at 10 estuary sites) confirmed 12S eDNA amplification of one or more marine fish species in all samples.

### New 12S reference sequences

The pilot study described above generated a number of sequences with absent, incomplete, or non-exact GenBank matches. Based on these findings and our checklist of local fish, we sought tissues of relevant species. 31 specimens representing 18 species were obtained from fisheries researchers, local fish stores, or bait shops (S8 Table). Reference specimens were sequenced for an approximately 725 bp fragment of 12S that encompasses the eDNA target site and for 648 bp COI barcode region, the latter to confirm species identification. Genetic identification of reference specimens was desirable because some were fin clips or filets, thus identifications could not be morphologically verified. The newly generated COI barcode region sequences were matched to existing reference sequences in GenBank using BLAST and in BOLD using BOLD ID engine. Species-level assignments with COI barcodes were possible because 1) most local marine fish species have COI reference sequences in GenBank or BOLD (whereas the representation of fish 12S sequences in the databases is much sparser) and 2) most fish species have diagnostic differences in this gene fragment. For those species which already had both COI and 12S sequences in GenBank, there were no differences in taxonomic assignments of reference specimens based on COI as compared to 12S. For amplification of 12S, M13-tailed versions of primer pair 12S229F/12S954R were utilized with amplification parameters as described [47]. For COI barcode region, COI-3 fish primer cocktail and amplification parameters were as described [48]. Bidirectional sequencing using M13 primers was done at GENEWIZ. Sequences are deposited in GenBank (12S accession nos. KX686069-KX686099; COI, KX688296-KX688324).

### Collection sites

Sampling was done at two estuary locations with contrasting hydrology [18,49] (Figs 1 and 2). The East River site (40.760443, -73.956354) is a narrow (275 m), deep (8 m at shoreline to 30 m mid-channel), high flow (2.7 m/sec and 2.5 m/sec, on ebb and flood tides, respectively) tidal strait, with salinity and transparency close to seawater. The Hudson River location (40.794711, -73.978902) is more typical estuarine: broad (1325 m), sloping (1 m at shoreline to 14 m mid-channel), relatively low flow (1.4 and 1.0 m/sec on ebb and flood tides, respectively), with lower salinity (on average about one-half that of seawater) and higher turbidity than the East River.

### Sampling method and schedule

Water sampling in protected areas was collected under permit from New York City Department of Parks and Recreation. Fish specimens were collected under permit from New York State Department of Environmental Conservation by NYSDEC personnel, or were obtained from scientific collections at Monmouth University. All sampling procedures were approved as part of the field permit. No animals were housed or experimented upon as part of this study. No endangered or protected species were collected. One liter surface water samples were collected from the shoreline at both sites approximately weekly from January 2016 to July 2016. Assuming that many species would be difficult to detect, on most (60%) days a second collection was made on the opposite tide (approximately 6 h later) to maximize the chance of detection. Analyzing possible differences by tide would require many more samples at standardized points in the tidal cycle. Because the shoreline at the sampling sites is armored and elevated, water was collected with a bucket on a rope and transferred to two 500 mL plastic bottles (Fig 2).

### Filtration

All work described below including DNA analysis was performed on a benchtop, with a separate work area for post-PCR procedures. After collection, samples were filtered within 1 h or stored at 4°C for up to 72 h before filtration. Filtration apparatus consisted of a 1000 mL side arm flask attached to wall suction, a frittered glass filter holder (Millipore), and a 47 mm, 0.45 µM pore size nylon filter (Millipore). Before filtration, water was poured through two paper coffee filters (Melitta) to exclude large particulate matter. After filtration, nylon filters were folded to cover the retained material and stored in 15 mL conical tubes at -20° C until DNA extraction. As environmental controls, we filtered water samples from controlled or remote environments that do not share fish taxa with the Hudson estuary (New York Aquarium; Marco Island, FL). There was no evidence of cross-contamination of fish eDNAs, i.e., none of the fish eDNAs present in local samples were detected in controls and vice versa (S1 File). The results themselves support the validity of detections—consistent differences over time and between sites, and the congruence of eDNA and seining studies. This strategy was not informative as to the source(s) of human and domestic animal DNA, which was detected in samples from experimental and control environments—as frequently observed in eDNA studies [38].

### DNA extraction, amplification, and library construction

Filters were processed using MoBio Powersoil Kit with modifications from the manufacturer’s protocol to accommodate extraction from a filter as described in S9 Table. Following extraction, DNA concentration was measured using Qubit (Thermo Fisher Scientific) (typical yield 1–5 µg DNA/L water), samples were further purified with AMPure XP (Beckman Coulter) following manufacturer’s protocol, and resuspended in 50 µl of Elution Buffer (10 mM Tris, pH 8.3) (Qiagen).

To facilitate library construction, we adapted the Illumina 16S metabarcoding protocol, adding tails to 12S-V5 primers [29] described above. Primers were obtained from Integrated DNA Technologies with the following sequences (Illumina tails in italics):

Forward: 5’- *TCG TCG GCA GCG TCA GAT GTG TAT AAG AGA CAG* ACT GGG ATT AGA TAC CCC -3’. Reverse: 5’- *GTC TCG TGG GCT CGG AGA TGT GTA TAA GAG ACA G*TA GAA CAG GCT CCT CTA G -3’. The amplified target not including primers is approximately 110 bp; the entire amplicon including tailed primers is approximately 200 bp.

Amplifications were done with Illustra puReTaq Ready-To-Go PCR beads (GE Healthcare), 5 μl DNA (representing eDNA from 100 mL of water from estuary or control samples) or 5 μl H2O for negative PCR control, 200 nM each primer, in final volume 25 μl. Parameters were 95°C x 7 m, then 40 cycles of (95°C x 30 s, 52°C x 30 s, 72°C x 30 s), followed by 72°C for 10 m, and hold at 4°C. 5 μl of each reaction were run on a 2% agarose gel with SYBR Safe dye (Thermo Fisher Scientific) to visualize amplifications and confirm negative control. PCR products were treated with AMPure XP to remove unincorporated primers and nucleotides and resuspended in 40 μl Elution Buffer.

To enable pooling of libraries, Nextera index primers (Illumina) were added following manufacturer’s protocol, using 10 μl of 12S PCR product, 2.5 μl each primer, GE Illustra beads with final volume of 25 μl, and amplification parameters 95°C x 3 m, then 8 cycles of (95°C x 30 s, 55°C x 30 s, 72°C x 30 s), extension at 72°C x 5 m, hold at 4°C. 5 μl of each reaction were run on a 2% agarose gel with SYBR Safe dye to confirm amplification.

Indexed PCR products were treated with AMPure XP, resuspended in 40 μl Elution Buffer, and DNA concentration was determined with Qubit. A pooled sample containing 27 ng of each library at 15 nM was sequenced at GENEWIZ on an Illumina MiSeq (2 x 150 bp). Negative library controls as described above were included in each pool. The 76 experimental and 11 control libraries, plus samples from other studies not reported here, were analyzed in four MiSeq runs with 35–60 libraries per run.

To assess reproducibility, 42 DNA samples were re-amplified, indexed, and submitted for MiSeq sequencing, and a two-way comparison of each pair of amplifications was performed. Each taxon detection was classified by number of reads and detection of that taxon in the paired sample (S5 Table).

### Bioinformatics

The paired FASTQ files generated by the MiSeq instrument were analyzed using DADA2 [50]. DADA2 was chosen because it uses an error model to infer exact sample sequences that can vary by as little as a single nucleotide. This is an alternative to cluster-based methods that traditionally lump sequences at 3% identity. This technical detail is important because the 12S amplicon is short (~100bp not including primers) and some fish species differ at only one or a few nucleotide positions; clustering would potentially lump such taxa together. DADA2 was used to merge paired FASTQ files and infer sequence variants using the default error model parameters, with one modification. We changed the sentence description of DADA2’s error model. The default behavior for DADA2 is to build an error model for each basepair for every fastq file that is provided. Alternatively, you can build an error model using a subset of the total reads from a sequencing run and provide this model to DADA2. We used the default value and have modified the description of this choice in the methods to read as follows:

“DADA2 was run in “self-consist” mode so that the error-model was independently built for each sample. This error model uses FASTQ quality scores to assess the likelihood at each base-pair that small mutations are due to true changes in the underlying biological sample rather than an error introduced during sequencing.” The primary outputs were a FASTA file of unique sequences (S2 File) and an operational taxonomic unit (OTU) table (S1 File) providing the abundance of each sequence in each experimental sample.

For each MiSeq run, OTU sequence counts were filtered to reduce library assignment errors. In pilot studies, we found misassigned reads in a given library for a given taxa were present on average at about 0.02% of total reads per taxa in the pooled library sample or 0.02% total reads per library. Misassignment classification was based on detection of species from non-overlapping environments (e.g., marine species in freshwater samples). Based on these results, in the present study we excluded detections representing less than 0.1% of reads per taxa or per library, which usually worked out to exclusion of read counts less than 100. Identical thresholds are applied in a recent aquatic eDNA study [25]; more detailed approaches to false-positive errors are recently proposed [51]. The average for non-excluded read counts was much higher, about 5,000. No fish species were entirely eliminated from a MiSeq run by these thresholds.

Environmental library controls were negative for estuary fish eDNAs after filtering (S1 File). Total reads per MiSeq run were roughly similar (about 10 M reads), regardless of the number of libraries in the run (35–60). We therefore considered that reads were relatively inflated in runs with fewer libraries. To enable compiling of results from different runs, we first chose 48 libraries per run as an arbitrary standard. OTU table reads were then adjusted accordingly (#reads x 48/#libraries in the run). Maximum adjustment between library runs was 1.7x. The adjustment did not change any presence/absence results and did not change the timing of eDNA increases for the three species in Fig 4. Alternate approaches are described [52]. The sequential modifications of each DADA2 OTU table are included in S1 File.

Species identifications were made in a multi-step process. Reads that were 100% identical to an internal library (comprised of GenBank 12S sequences for local species and the new 12S sequences generated in this study) were recorded. In the second step, all DADA2 unique reads (including those with 100% match to internal library) were submitted to BLAST engine in GenBank and results were screened for similarity (>90% identity) to vertebrate 12S sequences; those lacking similarity were excluded. About 1% of fish reads were minor variants (1 or 2 nucleotide differences) of 100% matching sequences present in the same run (for details see OTU tables in S1 File); these were excluded. Most of the minor variants were unique to a particular sample and library run—these likely represent sequence errors; others may be biological variants. Final species assignments were made using BLAST results, the checklist of local species, and fish distributions as recorded in FishBase. The identification process applied to all 76 libraries yielded 51 unique fish sequences. Most (45) were named based on 100% full-length identity to one or more reference sequences in GenBank or from this study; three cases were based on 99% identity; and there were three incomplete identifications (to genus or family level) based on 92%-99% identity.

As expected given the short fragment of 12S analyzed, some amplicons gave 100% full-length matches to multiple species. In some cases, a species-level identification could be made based on geographic range, i.e., only one of the matching species is present in the northwest Atlantic. In two cases (herrings, hakes), the amplicon sequence matched multiple local species. These detections were assigned to genus or family level, based on the lowest taxonomic group that comprised all matches. Identification details for individual sequences are in S4 Table. Species identifications including DNA sequences are listed in in S4 Table. Original fastq files with metadata are deposited in NCBI Sequence Read Archive (NCBI BioProject ID PRJNA358446).

### Statistics

Statistical analysis was performed using GraphPad Prism7 software.

## Supporting information

S1 Table Lower Hudson River estuary checklist and eDNA detection. (XLSX)

S2 Table Statisical analysis of fish eDNA detection vs checklist abundance. (XLSX)

S3 Table Fish eDNA reads by sample. (XLSX)

S4 Table Fish eDNA identification. (XLSX)

S5 Table eDNA detection reproducibility with repeat PCR. (XLSX)

S6 Table Non-fish vertebrate eDNA. (XLSX)

S7 Table Primer evaluation *in silico*. (XLSX)

S8 Table New 12S, COI sequences this study. (XLSX)

S9 Table Modified MOBIO PowerSoil DNA extraction protocol. (XLSX)

S1 File DADA2 OTU tables with analysis. (XLSX)

S2 File Compiled DADA2 FASTA files. (FAS)

S3 File Bioinformatic pipeline software. (R)
